# Assessing the Role of Uric Acid as a Predictor of Preeclampsia

**DOI:** 10.3389/fphys.2021.785219

**Published:** 2022-01-13

**Authors:** Ana I. Corominas, Yollyseth Medina, Silvia Balconi, Roberto Casale, Mariana Farina, Nora Martínez, Alicia E. Damiano

**Affiliations:** ^1^Hospital Nacional Profesor Alejandro Posadas, Buenos Aires, Argentina; ^2^Laboratorio de Biología de la Reproducción, Instituto de Fisiología y Biofísica Bernardo Houssay (IFIBIO) - Consejo Nacional de Investigaciones Científicas y Técnicas (CONICET) - Facultad de Medicina, Universidad de Buenos Aires, Buenos Aires, Argentina; ^3^Laboratorio de Fisiopatología Placentaria, Centro de Estudios Farmacológicos y Botánicos (CEFyBO) - CONICET, Universidad de Buenos Aires, Buenos Aires, Argentina; ^4^Departamento de Ciencias Biológicas, Cátedra de Biología Celular y Molecular, Facultad de Farmacia y Bioquímica, Universidad de Buenos Aires, Buenos Aires, Argentina

**Keywords:** uric acid, diagnostic value, biomarker, preeclampsia, intrauterine growth restriction (IUGR)

## Abstract

We assessed the diagnostic utility of uric acid for the prediction of preeclampsia. An observational prospective approach was carried out during 2014. Preeclamptic women were classified into 4 groups accordingly to the onset of preeclampsia and the presence of intrauterine growth restriction (IUGR). Serum uric acid levels, urea, and creatinine were measured. Receiver operating curves (ROC) of the uric acid levels ratio (UAr) between a dosage before and after the 20th week of gestation were performed. One thousand two hundred and ninety-third pregnant women were enrolled in this study. Eight hundred ten had non-complicated pregnancies, 40 preeclampsia, 33 gestational hypertension, and 20 IUGR without preeclampsia. Uric acid significantly raised after 20 weeks of gestation in women who develop preeclampsia before 34 weeks (Group A) or in those who develop preeclampsia after 37 weeks associated with IUGR (Group C). In women who develop preeclampsia after 34 weeks without IUGR (Groups B and D), uric acid increased after the 30th week of gestation. In all groups, UAr was greater than 1.5. In gestational hypertension, UAr was superior to 1.5 toward the end of gestation, while in IUGR without preeclampsia, the behavior of serum uric acid was similar to non-complicated pregnancies. In all cases, urea and creatinine showed normal values, confirming that patients had no renal compromise. ROC area was 0.918 [95% confidence interval (CI): 0.858–0.979) for the preeclampsia group and 0.955 (95% CI: 0.908–1.000) for Group A. UAr at a cut-off point ≥1.5 had a very low positive predictive value, but a high negative predictive value of 99.5% for preeclampsia and it reached 100% for Group A. Thus, a UAr less than 1.5 may be a helpful parameter with a strong exclusion value and high sensitivity for those women who are not expected to develop preeclampsia. Additionally, this low-cost test would allow for better use of resources in developing countries.

## Introduction

Preeclampsia is a multisystem disorder of unknown etiology that is unique to human pregnancy. Its clinical presentation is highly variable, and it complicates 10 million pregnancies annually, resulting in 76,000 maternal and 500,000 fetal or newborn deaths worldwide. In Latin America, its incidence is between 5 and 7% and it is the leading cause of maternal death (∼26%) while in Argentina, hypertensive disorders of pregnancy are responsible for 12.4% of maternal deaths (Abalos et al., 2013; Giachini et al., 2017; Guevel, 2018).

The consequences of this syndrome are not limited to pregnancy. They can lead to permanent vascular and metabolic damage and an increased risk of developing cardiovascular diseases in both the mother and the offspring (Huda and Greer, 2011; Abalos et al., 2014; Giachini et al., 2017). Although this pathology cannot be cured its early detection and referral to a high-complexity center are crucial to minimizing pregnancy complications and the long-term consequences. However, up to now, women who develop preeclampsia are only diagnosed after the onset of clinical symptoms, which makes patient management much more complicated.

Because of the serious consequences of this syndrome, it is still a challenge to predict which women are at risk of developing preeclampsia. So far, several promising biomarkers that could be used to make an early diagnosis have been identified. Anti-angiogenic factors such as soluble fms-like tyrosine kinase-1 (sFlt-1) and soluble endoglin (sEng), as well as pro-angiogenic factors such as vascular endothelial growth factor (VEGF) and placenta growth factor (PIGF) have demonstrated some benefit in the prediction or diagnosis of preeclampsia, as well as in the understanding of its etiology (Huppertz, 2018; McCarthy et al., 2018; Baert et al., 2021; Dröge et al., 2021; Lim et al., 2021). However, the choice of a biomarker should not only be related to its biological characteristics and its participation in the pathogenesis of the disease, but also to the feasibility of being used as a screening tool. A marker that requires complex technology and does not show a positive cost-benefit would be inapplicable. This could be even more relevant in low-income countries, where the number of deaths related to hypertensive pregnancies is higher than in developed countries (Giachini et al., 2017).

Although an association between elevated serum uric acid levels and preeclampsia has been known since the beginning of the 20th century and although this increase is considered, by many authors, as a marker of the severity of the disease, the clinical utility of this knowledge is still debated (Slemons and Bogert, 1917; Corominas et al., 2014; Pecoraro and Trenti, 2020; Zhao et al., 2021).

In uncomplicated pregnancies, serum uric acid concentrations decrease 25–35% due to the pregnancy-induced expansion of the blood volume, the increase in renal blood flow, the glomerular filtration rate, and the uricosuric action of estrogen (Nicholls et al., 1973; Lind et al., 1984; Carter and Child, 1989; Martínez-Gascón et al., 2016). Later in pregnancy, the serum uric acid levels rise until the end of pregnancy, as a consequence of the increase in fetal production, the decreased binding to albumin, and the decline in renal clearance (Lind et al., 1984; Nwagha et al., 2009; Amini et al., 2014).

In preeclamptic pregnancies, the increase in serum uric acid levels may be related to decreased uric acid excretion (Roberts et al., 2005; Khaliq et al., 2018). However, the uricemia increase precedes the proteinuria increase (Corominas et al., 2014). More recently, the increased oxidative stress and the formation of reactive oxygen species were proposed as other contributing sources of the hyperuricemia observed in preeclampsia (Many et al., 1996; Bainbridge and Roberts, 2008; Masoura et al., 2015).

On the other hand, due to the uric acid interaction with proinflammatory cytokines, increased levels of uric acid in the plasma of patients with preeclampsia may indicate a direct contribution to the pathophysiology of this syndrome by its ability to promote inflammation (Mulla et al., 2011; Matias et al., 2015; Shirasuna et al., 2020).

However, the clinical significance of serum uric acid concentrations in monitoring hypertension in pregnancy is either minimally predictive or not predictive. A possible explanation is that the dosages were generally carried out at the beginning of gestation or after the onset of the clinical manifestations (Thangaratinam et al., 2006; Bellomo et al., 2011; Corominas et al., 2014; Chen et al., 2016; Pecoraro and Trenti, 2020).

Previously, we found that uric acid levels in preeclamptic pregnant women increased by at least 1.5 times after the 20th week of gestation, with no changes in uremia or creatininemia, showing the absence of renal compromise. We proposed that a Uric acid ratio (UAr) greater than 1.5 may be related to preeclampsia (Corominas et al., 2014). However, its predictive value is still under discussion.

Here, we propose to study the uric acid behavior throughout gestation to evaluate its predictive value to define the risk of developing preeclampsia.

## Materials and Methods

### Subjects

An observational prospective study was conducted to examine the behavior of serum uric acid levels throughout pregnancy and to ascertain its predictive value in determining the risk of developing preeclampsia. The study was approved by the Institutional Review Board and written consent was obtained from all subjects.

This study was carried out between January to December 2014, at the “Hospital Nacional Profesor Alejandro Posadas,” Buenos Aires, Argentina.

During this period, 1,293 pregnant women who received full antenatal care at the hospital were enrolled in this study. All women belonged to the white Hispanic ethnic group.

Previously, we have reported that uric acid levels did not change before the 20th week of gestation in women who developed preeclampsia, while in the second half of pregnancy these levels abruptly increased (Corominas et al., 2014). Consequently, we divided pregnancy into 4 stages to analyze the uric acid increase throughout pregnancy:

**Stage 1:** before 20 weeks of gestation

**Stage 2:** between 20 and 30 weeks of gestation

**Stage 3:** between 31 and 34 weeks of gestation

**Stage 4:** after 35 weeks of gestation.

In addition, preeclamptic pregnant women were stratified into 4 groups according to the onset of preeclampsia and the presence of low birth weights (Myatt et al., 2014):

**Group A:** women who develop preeclampsia before 34 weeks

**Group B:** women who develop preeclampsia between 34 and 36.9 weeks

**Group C:** women who develop preeclampsia after 37 weeks with fetal growth restriction

**Group D:** women who develop preeclampsia after 37 weeks without low birth weight.

Serum samples from each pregnant woman were collected throughout pregnancy and stored at −80°C until analyzed.

The gestational age of the patients at the time of collection was calculated as the time between the first day of the last menstrual period and the date of the blood analysis.

Uncomplicated gestations were defined as healthy pregnancies, with no underlying maternal condition that could adversely affect the pregnancy.

Gestational hypertension and preeclampsia were defined based on the FLASOG (Federacion Latinoamericana de Sociedades de Obstetricia y Ginecología) guidelines.

Gestational hypertension was defined as diastolic blood pressure persistently ≥140 mmHg and/or ≥90 mmHg diastolic, on two occasions (at least 6 h apart), after the 20th week of gestation without proteinuria, in a previously normotensive woman.

Preeclampsia was defined as systolic blood pressure ≥140 mmHg and/or diastolic pressure ≥90 mmHg, with proteinuria ≥0.3 g/day or 2 pluses on urine dipstick after the 20th week of gestation in a previously normotensive patient.

Intrauterine growth restriction (IUGR) was defined as a fetal birth weight lower than the 10th percentile for the gestational age. Gestational age-specific birth weight centile was based on data provided by WHO^1^.

Women who carried on multiple pregnancies, women with a diagnosis of chronic kidney disease, chronic hypertension, liver disease, collagen vascular disease, diabetes, major fetal abnormalities, cardiovascular disease, cancer, and those who declined to take part in the study were all excluded.

### Serum Uric Acid Quantification

Serum uric acid was measured using a color-enzymatic diagnostic kit by Roche (Buenos Aires, Argentina) in a Hitachi 917 analyzer according to the manufacturer’s protocol. Reference values for women are 2.4–5.7 mg/dL.

### Serum Urea and Creatinine Quantification

Serum urea was measured by an enzymatic diagnostic kit supplied by Roche (Buenos Aires, Argentina) in a Hitachi 917 analyzer according to the manufacturer’s protocol. Reference values are 0.10–0.50 mg/dL.

Serum creatinine was assessed by a colorimetric-kinetic kit based on the Jaffe reaction supplied by Roche (Buenos Aires, Argentina) in a Hitachi 917 analyzer according to the manufacturer’s protocol. The reference normal values are 0.5–0.9 mg/dL.

### Pre-pregnancy Body Mass Index

BMI [weight (kg)/height (m)^2^] was based on measured height and maternal report of pre-pregnancy weight.

### Statistical Analysis

We analyzed the data with two sample *T*-tests for equal variances, and with Wilcoxon signed-rank test when data were not normally distributed.

Multiple comparisons were evaluated by one-way analysis of variance (ANOVA test) followed by Fisher LSD test.

Association between UAr and BMI, UAr and parity, and UAr and maternal age were checked by chi-square test. Phi coefficient, Cramer’s V, and the contingency coefficient (Karl Pearson) were used to investigate the degree of association, excluding the effect of sample size. Correlation coefficients from 0 to 0.25 indicate little dependency and higher than 0.6 show great dependency.

Receiver operator characteristic (ROC) curves were performed to determine the optimum cut-off value and to evaluate the diagnostic accuracy.

Receiver operator characteristic curves of sensitivity vs. 1-specificity were plotted. This screening test is considered useless if the area under the curve (AUC) is less than 0.5. A screening test’s performance is considered good if the AUC is between 0.7 and 0.8, excellent if the AUC is between 0.8 and 0.9, and outstanding if the AUC is >0.9.

All statistical analyses were performed using the Statistix™ software version and the criterion of significance was *P* < 0.05.

## Results

Of the 1,293 pregnant women who received full antenatal care at the hospital enrolled in this study, only 1,256 were single pregnancies. Among these women, 810 were normotensive pregnant women without any other type of pathology, 40 presented preeclampsia and 33 gestational hypertension, and 20 IUGR without preeclampsia.

Tables 1, 2 show the clinical characteristics of the patients included in this study.

**TABLE 1:** Clinical Characteristics of the studied population.

	Uncomplicated pregnancies	Preeclampsia	*Gestational hypertension*	IUGR without preeclampsia
*n*	810	40	33	20
Maternal age (years)	24.94 ± 6.41	26.6 ± 6.9	29.87 ± 9.07	25.95 ± 7.59
Gestational age (weeks)^1^	38.72 ± 1.33	36.75 ± 2.88	38.1 ± 2.2	35.8 ± 3.75
Birth weight (g)	3320.9 ± 455.6	2871.6 ± 968.2	2978.0 ± 814.3	2091.8 ± 569.23***
Body mass index (BMI), kg/m2	25.1 ± 5.9	28.5 ± 7.9	29.21 ± 18.9	23.03 ± 5.54
Sistolic blood pressure (mmHg)	110.0 ± 4.1	158.6 ± 6.7***	157.0 ± 4.3***	116.2 ± 3.5
Diastolic blood pressure (mmHg)	63.1 ± 2.5	107.0 ± 3.8***	101.1 ± 9.4***	67.3 ± 6.2
Proteinuria^2^	*Negative*	+	*Negative*	*Negative*

*Values are mean ± SD.*

***P < 0.001 compared to uncomplicated pregnancies.

*^1^Weeks from last menstrual period.*

*^2^Proteins in urine were determined by Test Urine Labstix Strip.*

**TABLE 2:** Characteristics of studied preeclamptic population accordingly the different presentations of preeclampsia.

Preeclampsia	*n*	Gestational age (weeks)	Birth weight (g)
Group A Gestational age <34 wks	5	30.19 ± 2.48	1440.6 ± 737.4
Group B Gestational age between 34 and 36.9 wks	13	35.44 ± 0.78	2331.5 ± 501.0
Group C Gestational age >37 wks with fetal growth restriction	5	37.8 ± 0.75	2405.8 ± 94.7
Group D Gestational age >37 wks without fetal growth restriction	17	38.48 ± 0.96	3276.7 ± 482.7

*Values are mean ± SD.*

**FIGURE 1:**
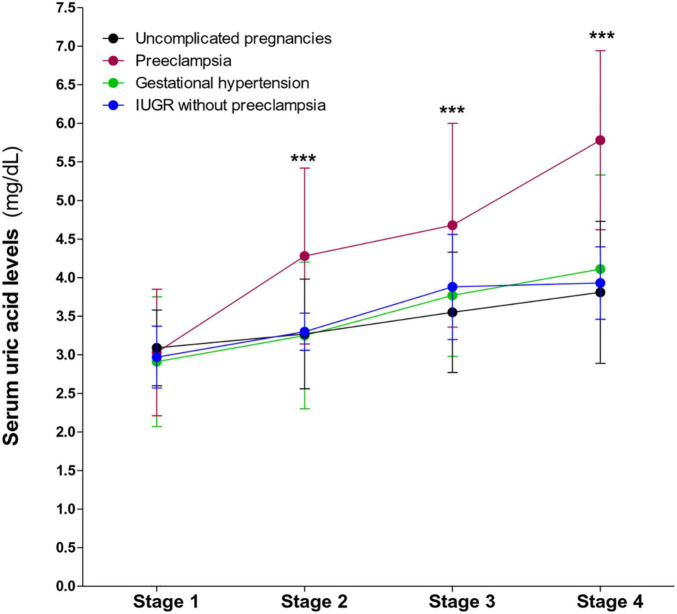
Behavior of serum uric acid levels throughout gestation in preeclampsia, gestational hypertension, IUGR without preeclampsia, and in non-complicated pregnancies. A significant increase in uric acid levels is observed in preeclampsia. Values are mean ± SD. ***P < 0.001 preeclampsia vs. uncomplicated pregnancies.

First, we evaluated the serum uric acid levels during gestation. As previously described, serum uric acid levels in preeclamptic women significantly increased, compared to uncomplicated pregnancies, until the end of pregnancy. In women with gestational hypertension uric acid levels tended to rise toward the 35th week of gestation, however, this increase is not statistically significant (*P* = 0.068). In contrast, the uric acid levels in women who presented IUGR without preeclampsia were similar to those observed in non-pathological pregnancies (Figure 1).

No association was found between UAr and BMI (*p* = 0.404) and UAr and parity (*p* = 0.409). Although the interaction between UAr and maternal age was statistically significant (*p* = 0.011), Phi coefficient (0.092), Cramer’s V (0.092), and the contingency coefficient (0.091) showed no association between the UAr and maternal age.

Interestingly, the serum urea and creatinine dosages were normal throughout pregnancy in all the groups (Table 3). Therefore, renal failure may not be responsible for the increase in uric acid in preeclamptic pregnant women.

**TABLE 3:** Serum urea and creatinine levels in the studied population.

	*n*	Creatinine levels (mg/dL)		Urea levels (mg/dl)	
		Before 20 wks of gestation	After 20 wks of gestation	Before 20 wks of gestation	After 20 wks of gestation
Uncomplicated pregnancies	810	0.52 ± 0.09	0.50 ± 0.09	0.18 ± 0.06	0.17 ± 0.07
Preeclampsia	40	0.54 ± 0.12	0.56 ± 0.13	0.17 ± 0.06	0.20 ± 0.07
Gestational hypertension	33	0.55 ± 0.12	0.54 ± 0.19	0.16 ± 0.07	0.15 ± 0.05
IUGR without preeclampsia	20	0.57 ± 0.11	0.63 ± 0.23	0.18 ± 0.05	0.20 ± 0.06

*Values are mean ± SD. In all cases, serum urea and creatinine levels did not change throughout gestation (P > 0.05).*

We also analyzed the uricemia ratio and as we expected, it was >1.5 in women with preeclampsia. We also observed that UAr was also >1.5 in women who had gestational hypertension (Table 4), suggesting a link between uric acid and high blood pressure.

**TABLE 4:** Uric acid ratio (UAr) in uncomplicated pregnancies, preeclampsia, gestational hypertension, and IUGR without preeclampsia.

	Uric acid ratio (UAr)		
	Stage 2/1	Stage 3/1	Stage 4/1
Uncomplicated pregnancies	1.12 ± 0.02	1.22 ± 0.02	1.31 ± 0.03
Preeclampsia	1.63 ± 0.18	1.79 ± 0.21	2.17 ± 0.22
*Gestational hypertension*	1.32 ± 0.18	1.49 ± 0.17	1.67 ± 0.22
IUGR without preeclampsia	1.14 ± 0.07	1.36 ± 0.02	1.37 ± 0.11

*Uric acid ratio was >1.5 in women with preeclampsia since the early stages of gestation. In women with gestational hypertension, UAr was >1.5 at the end of gestation. In non-complicated pregnancies and IUGR without preeclampsia, UAr was <1.5 during all gestation. Values are mean ± SEM.*

Then, we deconstructed the group of preeclamptic pregnant women accordingly to the onset of the clinical manifestations. We found that Groups A and C, which are the most severe presentations of preeclampsia, showed drastic increases in serum uric acid levels between 20 and 30 weeks of gestation with a UAr greater than 1.5. On the other hand, Groups B and D showed a significant increase in uric acid levels after 31 weeks of gestation resulting in UAr higher than 1.5 (Table 5).

**TABLE 5:** Uric acid ratio (UAr) in women who developed different presentations of preeclampsia.

	Uric acid ratio (UAr)		
	Stage 2/1	Stage 3/1	Stage 4/1
Uncomplicated pregnancies	1.12 ± 0.02	1.22 ± 0.02	1.31 ± 0.03
Preeclampsia Group A	1.85 ± 0.47	2.33 ± 0.47	–
Preeclampsia Group B	1.30 ± 0.26	1.91 ± 0.38	1.86 ± 0.26
Preeclampsia Group C	1.83 ± 0.56	1.96 ± 0.58	2.38 ± 0.57
Preeclampsia Group D	1.51 ± 0.28	1.70 ± 0.28	2.07 ± 0.34

*Groups A and C showed UAr >1.5 during all gestation. On the other hand, Groups B and D showed UAr >1.5 after 30 weeks of gestation. Values are mean ± SEM.*

Then, we compared uric acid levels in IUGR pregnancies with and without preeclampsia. Interestingly, uric acid levels only increased when the fetal growth restriction was associated with preeclampsia showing an UAr greater than 1.5 (Table 6).

**TABLE 6:** Uric acid ratio (UAr) in IUGR pregnancies with or without preeclampsia.

	Uric acid ratio (UAr)		
	Stage 2/1	Stage 3/1	Stage 4/1
IUGR without preeclampsia	1.14 ± 0.07	1.36 ± 0.02	1.37 ± 0.11
IUGR with preeclampsia	1.84 ± 0.27	1.97 ± 0.33	2.44 ± 0.41

*In IUGR pregnancies without preeclampsia, UAr was <1.5 along gestation. In contrast, in IUGR pregnancies with preeclampsia, UAr was higher than 1.5 since the early stages of gestation. Values are mean ± SEM.*

**FIGURE 2:**
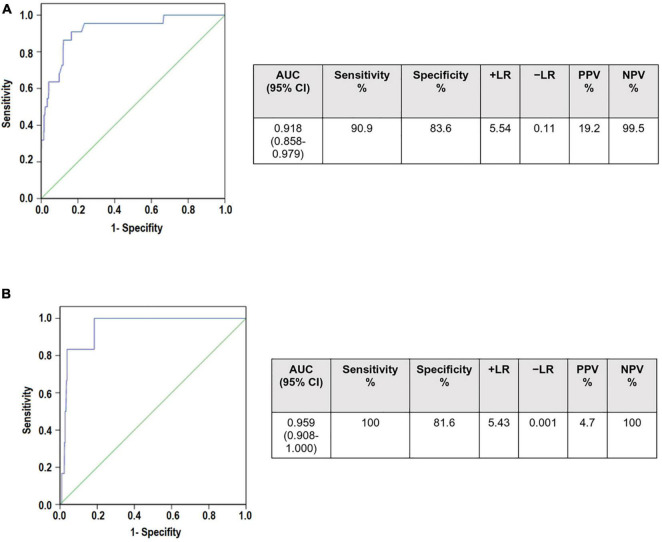
Receiver operating curves (ROC) curves to determine the diagnostic utility of serum uric acid ratio in **(A)** Preeclampsia **(B)** Preeclampsia Group A (who develop preeclampsia before 34 weeks of gestation). AUCs for both curves were >0.9 showing that UAr may be a good screening tool. However, it has very low positive predictive values (19.2 and 4.7, respectively). In contrast, the negative predictive values were 99.5% for preeclampsia and 100% for Group A of preeclampsia. Area under the curve (AUC), confidence interval (CI), Positive Predictive Values (PPV) Negative Predictive Values (NPV), probability indices (LR).

Finally, we studied the diagnostic performance of serum uric acid as a biomarker of preeclampsia. ROC curves were constructed with uricemia ratios (uricemia after the 20th week of gestation/uricemia before the 20th week of gestation) in patients with preeclampsia and normotensive pregnant women without any pathology (Figure 2A). Sensitivity and specificity were calculated for each cut-off point. The cut-off point with maximum sensitivity and specificity was equal to 1.50. With these data, the area under the curve (AUC), the Positive Predictive Values (PPV) Negative Predictive Values (NPV), and the probability indices (LR) were calculated. We also contrasted ROC curves for the Group A of preeclampsia in which the clinical manifestations occur before 34 weeks of gestation (Figure 2B).

Our results showed the AUC was 0.918 [95% confidence interval (CI): 0.858–0.979) for the preeclampsia group and 0.955 (95% CI: 0.908–1.000) for Group A of preeclampsia, showing that the uricemia ratio can be considered as a screening test. However, a UAr at a cut-off point ≥1.5 had a very low positive predictive value. Interestingly, it had a high negative predictive value of 99.5% for preeclampsia and it reaches 100% for Group A of preeclampsia.

## Discussion

Hypertensive disorders of pregnancy are a relevant medical problem that affects a high number of pregnant women, being associated with the development of both maternal and fetal complications. Within these disorders, preeclampsia constitutes the most severe of the hypertensive complications of pregnancy.

Currently, many molecules have been proposed as biomarkers for preeclampsia, although their usefulness and versatility are controversial. Among them, angiogenic markers such as s-Eng or s-Flt-1/PlGF ratio have been demonstrated to be the most promising (Verlohren et al., 2012; Brownfoot et al., 2017; Dröge et al., 2021). Unfortunately, the clinical use of these biomarkers requires expensive and sophisticated technologies. The implementation of these tests as a routine medical practice in all pregnant women seems not to be feasible, particularly in developing countries of Latin America, Asia, or Africa, with a low-income socioeconomic environment.

On the other hand, the role of uric acid in preeclampsia has generated growing interest.

Although the link between increased uric acid levels and preeclampsia is well-known, the clinical value of this association is still up for discussion. The classical interpretation of the increase in serum uric acid levels proposes that vasoconstriction induced by hypertension causes a decrease in its renal clearance (Many et al., 1996).

With growing knowledge of the effects of uric acid on the endothelium, oxidative stress, and inflammation, features that are assumed to have a role in the pathogenesis of pre-eclampsia, the interest in the significance of uric acid as a prognostic marker of preeclampsia has been renewed (Mulla et al., 2011; Matias et al., 2015).

There are studies in the literature that support the use of uricemia as a predictor of this pathology (Many et al., 1996; Roberts et al., 2005; Bellos et al., 2020), but there are also others that criticize it arguing that its positive diagnostic value is not optimal. Even more, it was also proposed that increased uric acid levels have been linked to poor maternal and fetal outcomes in several studies (Ugwuanyi et al., 2021; Zhao et al., 2021). Nevertheless, other studies stated that a high uric acid level is an unreliable predictor of maternal and fetal outcomes (Thangaratinam et al., 2006; Chen et al., 2016; Pecoraro and Trenti, 2020).

In this work, we observed that during uncomplicated pregnancies the values of uric acid levels decrease between 25 and 35% at the beginning of gestation, showing lower values than in non-pregnant women, and then it increases slightly and reach toward the end of gestation similar values to those of non-pregnant women. This behavior could be attributed to estrogens’ uricosuric action (Nicholls et al., 1973). Since uricemia levels rise toward the end of pregnancy, this uricosuric impact must be addressed by some regulatory mechanism or by an overproduction of this metabolite. This rise could be also explained by the inflammatory reaction that occurs when labor begins (Romero et al., 2018).

Because of the biological variability of uricemia, we have previously proposed the calculation of the UAr (serum uric acid levels after 20 weeks of gestation/uric acid levels before 20 weeks of gestation) as an analysis tool. We proposed that a ratio greater than 1.5 would be related to the onset of preeclampsia (Corominas et al., 2014). Here, we conducted a prospective study to evaluate the diagnostic value of this uricemia ratio as a predictive marker of preeclampsia. Although one of the limitations of our study was the low number of preeclamptic women included in the different subgroups, it is in concordance with the incidence of preeclampsia in our country (Corominas et al., 2014; Guevel, 2018).

We found that in all presentations of preeclampsia, serum uric acid levels increased. However, the time of the rise of uric acid levels depended on the severity of the disease. Thus, in women who develop preeclampsia before 34 weeks (Group A) or in those who develop preeclampsia after 37 weeks associated with IUGR (Group C), uric acid levels significantly raised after week 20 weeks of gestation. On the other hand, in women who develop preeclampsia after 34 weeks without IUGR, uric acid levels increased later in gestation (Groups B and C).

These findings proposed that the timing of the increase of serum uric acid and the magnitude of the increase (represented by the UAr) may be associated with the severity of the condition.

On the other hand, although in gestational hypertension uric acid levels showed a slight increase near to term, this rise was not statistically significant, possibly due to the small size of the sample. However, in these women, UAr was greater than 1.5 toward the end of gestation.

One of the major weaknesses of many of the potential biomarkers for preeclampsia is the difficulty in discerning between IUGR with preeclampsia and without preeclampsia (Litwińska et al., 2017; Huppertz, 2020). In this regard, our findings revealed that when the fetal growth restriction is not associated with preeclampsia, uric acid levels do not increase throughout pregnancy. Therefore, these results may support the hypothesis that the rise of uric acid levels is linked to maternal endothelial dysfunction and exacerbated systemic inflammatory response in preeclampsia (Huppertz, 2020; Redman et al., 2021). In this regard, we propose that monitoring uric acid levels during pregnancy, in combination with biochemical and ultrasonographic markers, could enable a better diagnosis of these disorders.

Based on ROC curves, we also demonstrated that the uricemia ratio (serum uric levels after 20 weeks of gestation/serum uric levels before 20 weeks of gestation) has diagnostic value. Regarding this, we found that a UAr less than 1.5 is a helpful parameter with a strong exclusion value and high sensitivity for those women who are not expected to develop preeclampsia. Even though it is uncommon to employ an analyte dosage as an exclusion tool, our results confirmed the clinical value of monitoring the uric acid levels during gestation as a “collaborator” in the identification and prediction of preeclampsia.

Although many studies have ruled out the value of uric acid as a predictor of preeclampsia in the first trimester, its evaluation in the second and third trimesters would still provide useful information to timely referring a woman at risk to a more complex center. The hunt for first trimester biomarkers does not solve the problem in our region, where about half of all women still go to their initial check-up after the first trimester (Corominas et al., 2014; Giachini et al., 2017).

In this sense, a marker that allows excluding those women who are not at risk of preeclampsia from those who potentially are would let us conduct a more exhaustive follow-up of these patients at risk and the opportune referral to a health center of more complexity. Thus, this would not only serve for the administration of effective prophylactic therapies to prevent the progression of the disease and improve perinatal obstetric outcomes but also for the minimization of the offspring’s long-term complications.

## Data Availability Statement

The raw data supporting the conclusions of this article will be made available by the authors, without undue reservation.

## Ethics Statement

The studies involving human participants were reviewed and approved by the Local Ethics Committee of the Hospital Nacional Profesor Alejandro Posadas, Buenos Aires, Argentina. The patients/participants provided their written informed consent to participate in this study.

## Author Contributions

AC and YM carried out the experimental work and analysis of data. SB, RC, MF, and NM carried out the data analysis and discussion and critically reviewed the manuscript. AD designed the study and wrote the manuscript. All authors contributed to the final version of the manuscript.

## Conflict of Interest

The authors declare that the research was conducted in the absence of any commercial or financial relationships that could be construed as a potential conflict of interest.

## Publisher’s Note

All claims expressed in this article are solely those of the authors and do not necessarily represent those of their affiliated organizations, or those of the publisher, the editors and the reviewers. Any product that may be evaluated in this article, or claim that may be made by its manufacturer, is not guaranteed or endorsed by the publisher.

## Footnotes

1
http://www.who.int/childgrowth/mgrs/en/
